# Characterization Methods to Determine Interpenetrating Polymer Network (IPN) in Hydrogels

**DOI:** 10.3390/polym16142050

**Published:** 2024-07-18

**Authors:** Ceren Cona, Katherine Bailey, Elizabeth Barker

**Affiliations:** Department of Mechanical, Aerospace, and Biomedical Engineering, University of Tennessee, Knoxville, TN 37996, USA; cbayar@vols.utk.edu (C.C.); kmbailey@utk.edu (K.B.)

**Keywords:** interpenetrating network, polymer, characterization, hydrogel

## Abstract

Significant developments have been achieved with the invention of hydrogels. They are effective in many fields such as wastewater treatment, food, agriculture, pharmaceutical applications, and drug delivery. Although hydrogels have been used successfully in these areas, there is a need to make them better for future applications. Interpenetrating polymer networks (IPNs) can be created to make hydrogels more adjustable and suitable for a specific purpose. IPN formation is an innovative approach for polymeric systems. It brings two or more polymer networks together with entanglements. The properties of IPNs are controlled by its chemistry, crosslinking density, and morphology. Therefore, it is necessary to understand characterization methods in order to detect the formation of IPN structure and to develop the properties of hydrogels. In recent studies, IPN structure in hydrogels has been determined via chemical, physical, and mechanical methods such as Fourier transform infrared spectroscopy (FTIR), Raman spectroscopy, scanning electron microscopy (SEM), field emission scanning electron microscopy (FESEM), differential scanning calorimetry (DSC), dynamic mechanical analysis (DMA), X-ray diffraction (XRD), and rheology methods. In this paper, these characterization methods will be explained, recent studies will be scrutinized, and the effectiveness of these methods to confirm IPN formation will be evaluated.

## 1. Introduction

Hydrogels are defined as three-dimensional and hydrophilic structures that consist of crosslinked networks [1]. Their swelling ability makes them perfect candidates for holding large amounts of water, biological substances, or other liquids. Hydrogels have been used successfully in many fields such as the food industry [2,3,4,5], agriculture [6], wastewater purification processes [7], and medical applications [8,9,10,11,12]. They have been especially impactful in biomedical applications. For instance, they are widely used in wound dressings [13], tissue engineering [13,14,15,16], sensors [17,18], and drug delivery [19,20,21].

Hydrogels can be synthesized from natural and synthetic polymers [22,23,24,25]. Synthetic polymers have a few advantages, such as tunable properties, prolonged stability in different environments, and being formed from various sources. However, they degrade into acidic waste products and may aggregate in the body. This may lead to foreign body response by the immune system and inflammation. In contrast, natural polymers provide suitable in vivo properties since they are known by the body. They can also be easily synthesized from natural sources in abundance [26]. Both synthetic and natural polymers are used in biomedical studies, and they have various benefits to the field. This paper focuses on hydrogels used in biomedical applications.

The network determines the hydrogel’s properties by influencing its softness/stiffness and ability to retain large amounts of liquid. Single network (SN) hydrogels appear to have limited loading capacity [4], and their mechanical properties usually make the hydrogel weak and soft [27,28]. Moreover, low-swelling SN hydrogels need an adjustment to make them more controllable for their applications [29,30]. Therefore, SN hydrogels sometimes do not meet the requirements for specific applications and need to be improved [3,31]. Since it is known that the majority of the properties of a hydrogel are determined by its networks, an advanced network structure may help to provide desired properties for the material [30]. An advanced interpenetrating polymer network (IPN) structure can be shown as a prospective form to upgrade the hydrogel’s properties.

**Figure 1:**
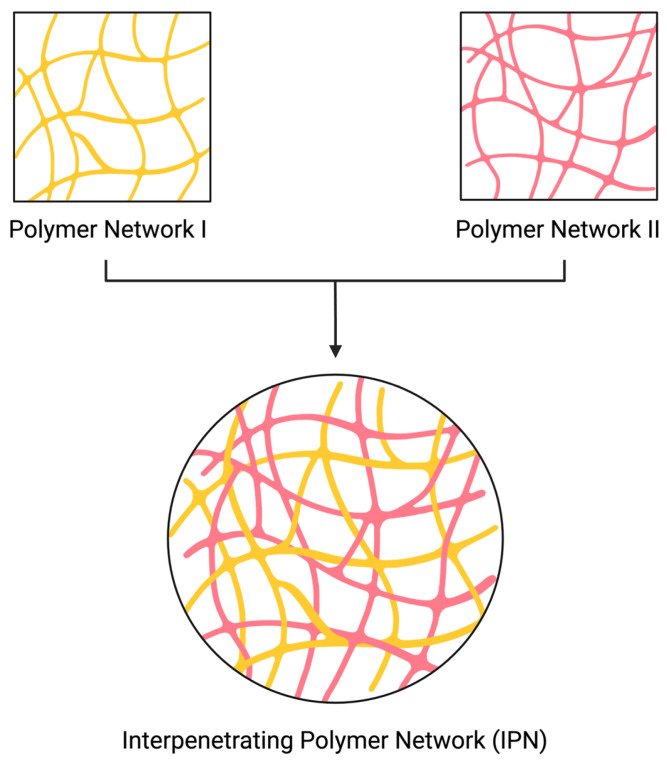
An illustration of IPN structure (made by BioRender.com).

**Figure 2:**
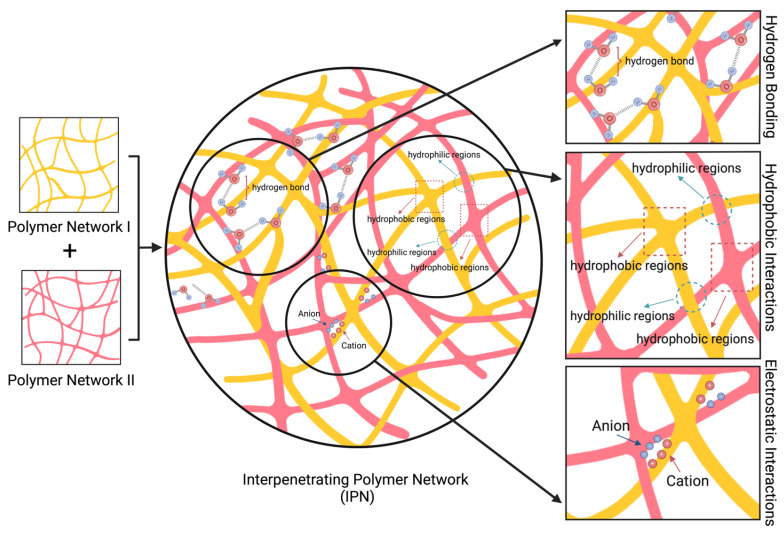
Physical forces that cause entanglements in IPNs (made by BioRender.com).

IPNs are polymeric structures that comprise two or more networks (Figure 1). These networks must be at least partially entangled on a molecular scale, but they cannot covalently bond with each other to be considered an IPN [4,32,33]. In most cases, networks entangle with physical bonding, such as electrostatic or hydrophobic interactions, van der Waals forces, and hydrogen bonding [33,34,35,36,37] (Figure 2).

**Figure 3:**
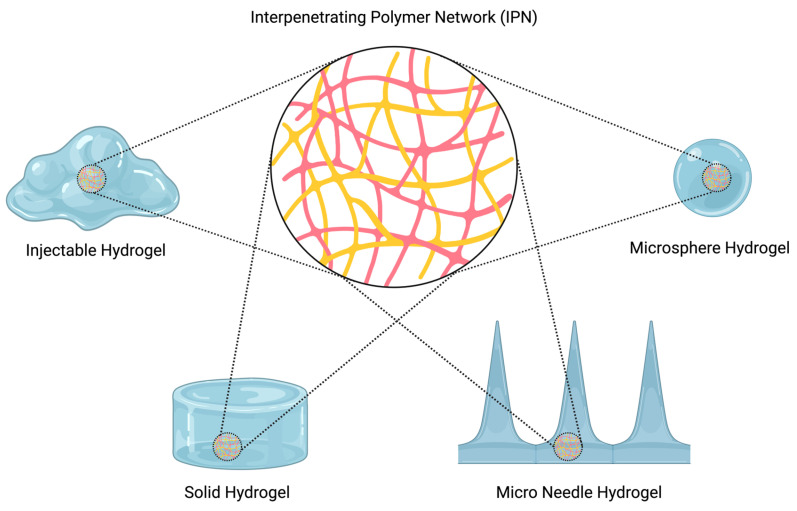
Different hydrogel forms of IPNs (made by BioRender.com).

IPN structure in hydrogels can be created in various forms such as films, beads, microspheres, nanoparticles, tablets, and gels [38] (Figure 3). Its structure can be altered as desired for various study fields. From a biomedical point of view, IPNs play an important role because of the gap between their networks. Due to this feature, they can be loaded with different types of drugs, living cells, and liquids [39]. They can even be formed by proteins to create internal reinforcement to structures such as tissue, organs, and systems [35,40]. The mechanical properties of IPNs can be conveniently altered as desired for a particular application. For instance, denser IPN forms may be created for support and reinforcement applications in tissue engineering, material science, and automotive and civil engineering. On the other hand, their structure is immensely practical for loading applications due to their ability to swell. The mechanical properties, such as the stiffness, toughness, and loading capacity, of hydrogels can be ameliorated by forming IPNs. In tissue engineering, wound dressing [39], and drug delivery applications, IPN technology is widely used to enhance treatment methods.

IPNs are usually classified into two main categories based on their formation method [24,40,41,42,43].

Sequential IPN: Formation of polymer network (PN) 1 occurs first. Then, the second PN with/without a crosslinker is polymerized into PN 1.

Simultaneous IPN: Constituent polymers are polymerized at the same time with/without crosslinker to form this kind of IPN.

The most common IPN structures obtained via these formation methods are semi-IPN and full-IPN; however, there are also other types of IPN.

Semi-IPN: Consist of one crosslinked network and additional, uncrosslinked polymer chains.

Full IPN: Consist of two individually crosslinked networks entangled with each other [44].

Pseudo-IPN: Pseudo-IPNs are a type of simultaneous IPN. One polymer has a crosslinked network while the other polymer is linear [44].

Gradient IPN: Gradient IPNs have macroscopically non-homogenous structures in different locations. To synthesize such an IPN, monomer A diffuses into polymer network B. Then, rapid polymerization occurs before diffusion equilibrium happens [45]. Gradient IPNs are collections of layers of insoluble polymer mixtures. Their characteristics can differ gradually in different locations in samples [46].

Thermoplastic IPN: These IPNs show thermoplastic behavior. Physical crosslinking can be used to form these networks. Thermoplastic IPNs behave like thermoplastics because their physical crosslinks break at higher temperatures [44].

IPNs help to bring together polymers that have different properties to form an advanced material. If a polymer or a polymer blend cannot achieve the desired properties, an IPN structure brings a different approach to the application by combining the features of different polymers. Hydrogels with novel properties can be synthesized for different applications with IPN structures. For instance, drug delivery via hydrogels shows great potential to achieve a suitable route to prevent and/or treat diverse diseases. As a novel drug delivery system, IPN hydrogels provide controlled drug release at the target site, minimizing exposure to the drugs at non-target sites so they decrease systemic toxicities and prevent side effects [41,47].

**Figure 4:**
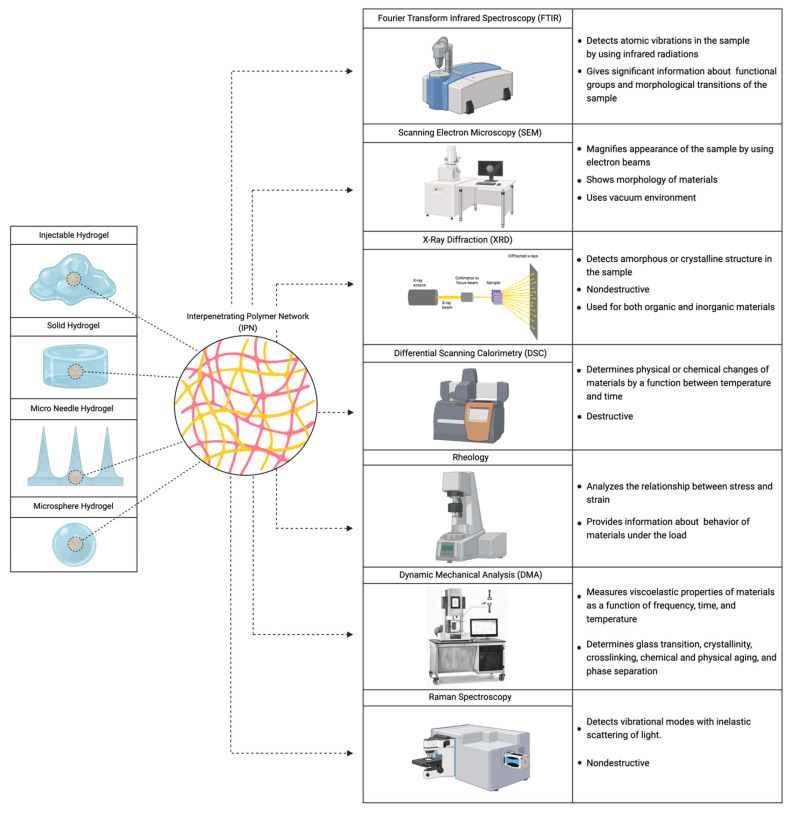
Characterization methods for IPN structures (made by BioRender.com).

Chemical, physical, and mechanical characterization methods have been conducted to understand the structure of the material and investigate whether it is an IPN or not. The most common characterization methods discussed in the literature are Fourier transform infrared spectroscopy, Raman spectroscopy, scanning electron microscopy, field emission scanning electron microscopy, differential scanning calorimetry, X-ray diffraction, dynamic mechanical analysis, and rheology [48,49]. Researchers use one or more characterization methods to assess the IPN structure of their material. However, these methods have not been brought together and explained in one paper (Figure 4).

In this paper, articles describing the different characterization methods to evaluate IPN structure are reviewed, interpretation of results explained, and an overall recommendation provided for which methods are most useful in proving the presence of IPN structures in hydrogels.

## 2. Characterization Methods to Determine the Formation of IPNs

First, characterization methods must be understood to analyze the formation of IPNs. A brief introduction to each of these methods is given below.

### 2.1. Spectroscopy Methods

**Figure 5:**
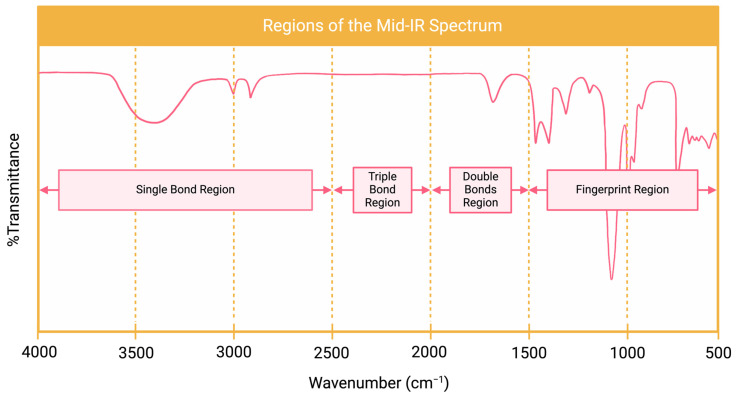
A general view of a mid-IR spectrum (made by Biorender.com).

Fourier transform infrared spectroscopy (FTIR) is used for the characterization of different kinds of materials by infrared radiation (IR). This method is an effective approach for materials/samples that can be present at solid, liquid, gas, film, fiber, and powder forms. It detects molecules’ atomic vibrations in the sample by using IR. FTIR is used in different applications such as drug and medical device development, food analysis, polymer science, industry, and academic research. It provides information about the chemical structure of molecules, functional groups and chemical bonding and chemistry identification/characterization of the sample [50]. Results from the FTIR analysis are understood by reading the FTIR spectrum. This spectrum shows “absorption values versus wavenumbers” or “transmission values versus wavenumbers”. To understand this spectrum, it is necessary to know in which region the analysis is performed. The infrared spectrum is separated into three main regions. These regions are the far-IR spectrum (<400 cm^−1^), mid-IR spectrum (400–4000 cm^−1^), and near-IR spectrum (4000–13,000 cm^−1^). The mid-IR spectrum is used mostly for the samples. Figure 5 shows a general view of a mid-IR spectrum.

According to Kumar et al. (2019), FTIR results provide significant information about materials’ functional groups and morphological transitions [51,52]. Earlier in this article, it was mentioned that physical entanglements between different networks form the IPN structure. Based on this information, the physical bonds between the networks can be found with the FTIR technique. This information may help researchers to interpret whether the IPN structure has occurred in a sample or not. In this article, some examples will be shown to demonstrate how researchers have used FTIR to determine and understand the presence of IPN structure in their studies.

Raman spectroscopy works in a similar manner to FTIR. The main differences between FTIR and Raman spectroscopy are working principle, sensitivity, and sample preparation. Raman spectrometry provides information about vibrational modes by measuring light. As mentioned in the FTIR section, FTIR works by absorbing IR light; however, Raman spectroscopy uses a laser, and its working principle is based on Raman scattering, which is the inelastic scattering of light. One of the most important feature of Raman spectroscopy is being less sensitive to water than FTIR. It is a nondestructive method and requires short preparation times [53]. According to Nigro et al. (2021) and Rajawasam et al. (2024), this method can be used as an identifier of IPN structures as in FTIR [32,54,55].

### 2.2. Electron Microscopies

Scanning electron microscopy (SEM) is used to examine materials with magnified appearance by using electron beams. This physical method generates 3D-like images that show the morphology of a material by creating a beam of electrons that move on the sample. Electrons do not pass through the sample, but they strike and reflect off a thin metal layer previously coated onto the sample. These electrons are captured by a sensor and evaluated by detectors to create an SEM image [56]. Also, with the help of an electron beam, the sample emits X-rays with unique energy, which are used to identify the material’s composition [57].

**Figure 6:**
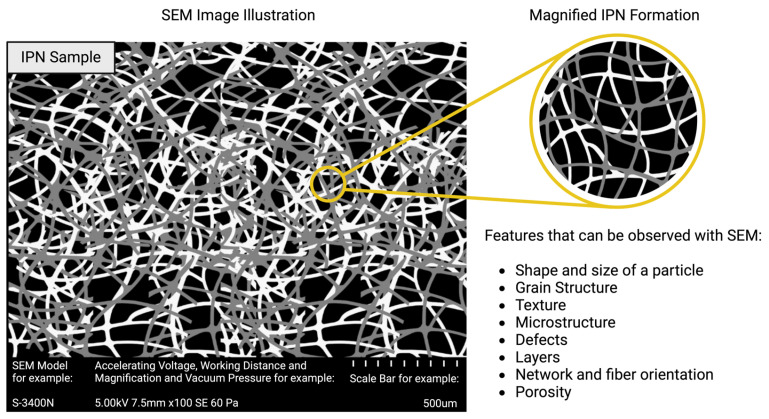
A SEM image illustration of an IPN sample (made by Biorender.com).

SEM is a nondestructive method that can detect a broad range of materials such as polymers, metals, ceramics, biological samples, and more within a vacuum environment [58,59,60,61,62]. SEM is an important method to determine IPN formation because it shows the arrangements, defects, voids, and structure of the material [63] (Figure 6). Therefore, the IPN structure can be observed and interpreted by using SEM, and these results might be used to prove whether or not an IPN has formed.

Similarly, field emission scanning electron microscopy (FESEM) can be used to observe the material’s morphologic properties and its IPN structure. It is convenient to use in the same applications as SEM, but it creates better quality images with higher sensitivity [64]. However, it also works under vacuum like SEM. If a vacuum environment cannot be formed, the presence of gas in the environment disorganizes the emitted electron beams, leading to inconclusive results [65]. The vacuum environment required for SEM and FESEM constricts their applications with materials such as gels, organisms, cells, and tissues.

Although these two methods seem very suitable for detecting IPNs, they have some drawbacks as mentioned before. SEM and FESEM require vacuuming and coating to examine samples [66], but hydrogels are swollen structures, so if they are vacuumed, their structure may change. For this reason, some researchers freeze-dry their hydrogel before using SEM. Freeze-drying helps keep the hydrogel in its swollen state and allows it to be separated into pieces to see the interior structure. For instance, Che et al. (2018) freeze-dried their hydrogel samples before examining with SEM [67]. This sample preparation method prevents the problems that can be caused by the vacuuming of the hydrogel. However, the freeze-drying method may create some structural changes in samples. Ice crystal formation can be observed with SEM after freeze-drying a hydrogel sample [68]. For these reasons, it is necessary to employ a characterization method other than SEM and FESEM in order to prevent undesirable effects from the vacuum environment and crystal formation.

Environmental scanning electron microscopy (ESEM) is a similar characterization method to see the magnified appearance of materials. It operates under normal/high-vacuum conditions, and pressure can be controlled when working on a sample. While the sample is exposed to atmospheric pressure, the electron column operates in a vacuum [69]. Moreover, it does not require coating of the sample [70]. This method allows researchers to investigate wet, damp, and biological samples without damage or altering their structure [71]. ESEM can be used as a suitable morphology observation method for IPN structure in hydrogels. Previous studies will be shown in the following parts to understand IPN structure observed from SEM, FESEM and ESEM images.

### 2.3. X-ray Diffraction

**Figure 7:**
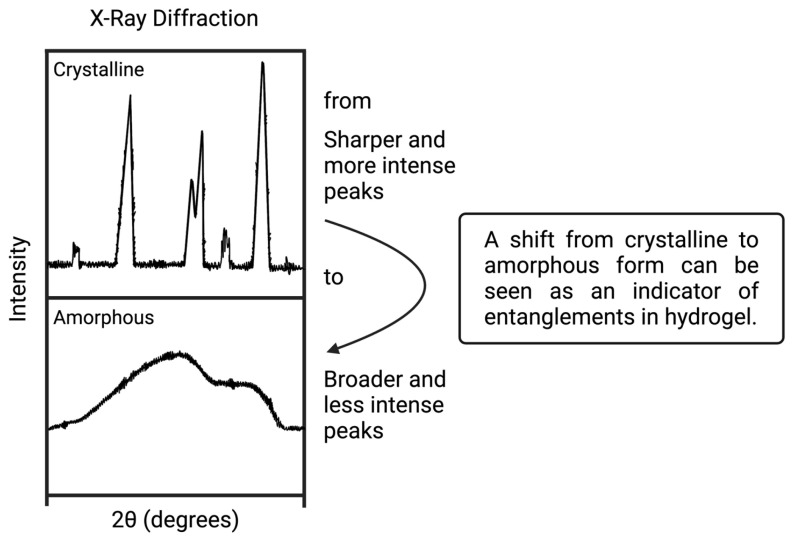
Crystalline and amorphous XRD pattern illustrations (made by Biorender.com).

X-ray diffraction (XRD) is a characterization method used to understand the crystal structure of a material [72]. Solid forms, liquids, and powders are examined with this technique. It is a nondestructive method and can be used for both organic and inorganic materials [73]. Briefly, X-ray beams pass into the sample, and they are deflected by the atoms of the sample. Then, diffraction patterns are gathered by a detector to show the sample’s crystallinity [74,75]. Diffraction patterns show the fingerprint of the material, provide information about the amorphous or crystalline structure of the material, and also measure the percent of crystallinity [76]. XRD characterization results can be useful for understanding IPN structure in hydrogels. Crystalline and amorphous structures provide information about the possibility of IPN formation within hydrogel (Figure 7).

### 2.4. Differential Scanning Calorimetry

Differential scanning calorimetry (DSC) determines physical or chemical changes of materials by a function between temperature and time [77,78]. This method requires small amounts of sample, and it can be used for both solid and liquid samples. However, it is a destructive method for materials due to heat [79]. DSC has two main parts: the thermal computer and a scanning chamber. Two pans are located in the scanning chamber for heating and cooling processes. The sample is settled within the first pan, and the other pan is left empty to use as a reference [75,80]. Temperature is changed gradually to produce a change in the material with the effect of heat quantity. Glass transition temperature (T_g_), crystallization heat (H_c_), melting point (T_m_), crystallization temperature (T_c_), and melting heat (H_f_) values are determined with DSC [81]. Water loss and thermal degradation studies can be performed for biopolymers. Also, according to Lu and Weiss (1992), T_g_ value is used to understand biopolymer blends’ miscibility [82,83]. Therefore, DSC can be used to analyze IPN structure in a hydrogel [84].

### 2.5. Dynamic Mechanical Analysis

Dynamic mechanical analysis (DMA) is a technique to measure the viscoelastic properties of materials as a function of frequency, time, and temperature. This technique is very similar to the rheology method. The differences between the rheology and the DMA methods are kind of the material, testing modes, and data output. Mostly, a rheometer is used for gel–liquid-like soft materials, while DMA is used for solid materials. Also, DMA tests include tension, compression, shear, and bending modes, but a rheometer is used to perform shear and normal stress analyses. DMA determines mechanical properties by the functions of temperature and time. Therefore, this method can be used for detecting morphological structures in a material. Glass transition, crystallinity, crosslinking, chemical and physical aging, and phase separation are used for the characterization of the material by DMA. The glass transition data indicate a relation to the viscoelastic response of the material. Viscoelastic materials, such as gels, show solid-like behavior at lower temperatures while they show gel-like or liquid-like behavior at higher temperatures [32,85,86,87,88,89]. Temperature or frequency can be kept constant while performing a DMA test; however, since it is usually difficult to work with very high and low frequencies, it is useful and fast to collect viscoelastic data with a constant frequency at different temperatures. The viscoelastic data can help to find viscoelastic transitions/relaxations in amorphous materials by the measured glass and secondary transition values. Since secondary transitions cannot be observed with DSC, DMA and DSC are sometimes used together for more accurate results. According to Chartoff et al. (2009), there is a relation between polymer crosslinking, network, and structure integrity with glass transition temperature [85]. This relation is used for characterizing network formation in materials in different applications such as polymer curing, filled polymers, pharmacological and biomedical device optimization studies, polymer composites, and wound healing applications [90,91,92,93,94].

### 2.6. Rheology

**Figure 8:**
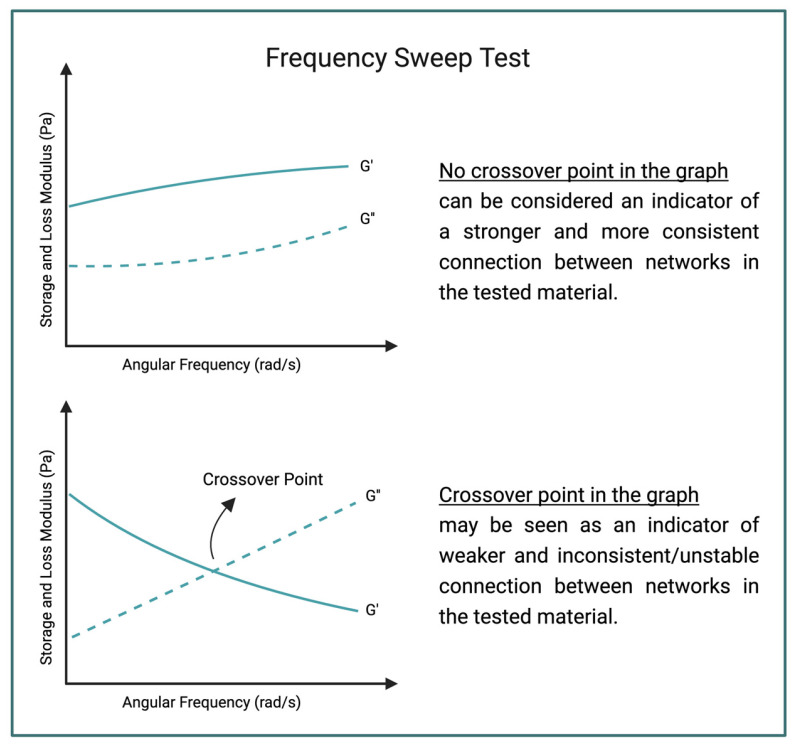
A generalized illustration of frequency sweep test graphs (made by Biorender.com).

Rheology is a term used for understanding viscoelastic behavior of a material. It analyses the relationship between stress (force) and strain (deformation) when a load is applied [95]. Examining hydrogels with rheology provides information about their behaviors under a load. Hydrogels can be shown as a complex system due to their liquid- and solid-like behaviors. For example, hydrogels used as injectable drug delivery devices display a shear thinning response and their structure can change with these applications. Therefore, it is important to understand the behaviors of injectable hydrogels for in vivo and clinical studies [96]. A rheometer is used to measure the mechanical behaviors of materials, and one of the tests which determine mechanical behavior is the frequency sweep test [97]. Figure 8 shows a generalized illustration of two different frequency sweep test graphs. According to Stojkov et al., crossover point is an indicator of consistent connections in a hydrogel [98]. These rheology tests may help determine IPN structure within a hydrogel [30,99]. Examples of using rheology to characterize IPN hydrogels will be covered in the next section.

## 3. Studies That Investigate IPN Formation

In this section, a detailed literature review of some IPN hydrogel articles will be discussed. Unfortunately, most of the available IPN studies do not indicate how they confirmed the IPN structure in the hydrogels, mentioning only that their hydrogel has an IPN structure without showing any concrete proof. The articles presented below actually try to prove the IPN structure in their material with different characterization methods.

### 3.1. Injectable Hydrogels

Wang et al. (2017) synthesized a dynamic covalent hydrogel which is injectable and has IPN structure. The purpose of their study was enhancing mechanical features and improving the structure of the gels for use as a novel approach in different applications such as 3D printing, tissue engineering, and drug delivery. They used reversible addition fragmentation chain transfer polymerization method to create a p(*N*, *N*-dimethyl-acrylamide-*co*-4-acryloyldopamine) p(*N*, *N*-dimethylacrylamide-*co*-3-acrylamidopheynlboronic acid) p(*N*, *N*-dimethylacrylamide-*co*-4-acryloyloxybenzaldehyde) gel. FTIR studies were performed at different pH values, and crosslinking in the gels was determined by peak disappearance within the 1700–1750 cm^−1^ band range, which was caused by -C=O and -NH-NH_2_ linkage. The mechanical properties of the gel (at pH 3, 7 and 10) were investigated by using rheometer frequency sweep tests. All the gel samples showed gel-like behavior (G′>G″). According to the authors, IPN hydrogels have higher strength values than single network hydrogels. Therefore, they suggested that the increased density of crosslinking leads to IPN and interlocked structure formation in the gel [100].

Shariatzadeh et al. (2021) synthesized an IPN cryogel which was formed from gelatin methacrylate (GelMA) and hyaluronic acid (HA) to be used for soft tissue engineering. An IPN gel was created by using the sequential method. First, GelMA was chemically crosslinked, then HA was physically crosslinked by using Fe^3+^ as a crosslinker agent, and ultimately these two networks formed an IPN cyrogel. Mechanical properties were investigated by using frequency sweep tests via rheometer. Samples showed gel-like behavior (G′>G″). FTIR study was performed with GelMA and GelMa-HA to investigate interactions in the material. According to authors’ findings, there was a slight shift in the FTIR bands in comparison with GelMA and GelMA-HA gels. This shift indicated electrostatic/intermolecular interactions or hydrogen bonding between HA and GelMA. Ultimately, it was interpreted as IPN formation in the GelMA-HA cryogel [101].

Cui et al. (2023) used a cellulose nanofiber (CNF) to create IPNs in oxidized alginate (OSA)/gelatin (Gel) hydrogel. The OSA/Gel/CNF hydrogel was formed to be used in bone repair studies because of its injectability, self-healing, and high strength properties. In this study, CNF acted as an IPN reinforcement to the base hydrogel. The effect of varying amounts of CNF (1%, 3% and 5%) to the hydrogel system was investigated by FTIR. Based on the FTIR results, the OSA/Gel had O-H stretching peak at 3521 cm^−1^, and it shifted to 3521 cm^−1^ when CNF3 was used to form OSA/Gel/CNF3. According to the authors, this shift at the O-H stretching band indicates the formation of hydrogen bonds. Moreover, this can be interpreted as a hint towards confirmed IPN structure in the OSA/Gel/CNF3 hydrogel [102].

Mu et al. (2020) synthesized an injectable IPN hydrogel which was formed by using injectable platelet-rich fibrin (iPRF) and gelatin nanoparticles (GNPs). The researchers created a hydrogel which had good mechanical strength, self-healing, and degradable properties. The purpose of the iPRF/GNP hydrogel use in bone healing studies as a bioactive material. Scanning electron microscopy was used to understand and opine regarding this double-network hydrogel’s structure. According to the study, the iPRF/GNP hydrogel showed a double-network structure in SEM images. Also, the authors asserted that the iPRF/GNP gel exhibited a porous network which was created from interconnected fibrin fibers and gelatin nanoparticles. They attributed this interconnection to cohesive interactions between these two components. These network formations confirm the IPN structure in the iPRF/GNP hydrogel [103].

Zhao et al. (2014) synthesized an injectable poly(ethylene glycol) methacrylate (PEGMA)/N-isopropylacrylamide (NIPAm)/methacrylated alginate (ALGMA) hydrogel for use in drug and protein release studies. The hydrogel was formed by a copolymerization method [104]. ALGMA was used in this study for its pH-sensitive and network-forming properties. The PEGMA/NIPAm gels with different ALGMA compositions were characterized via rheometer. The storage moduli of PEGMA/NIPAm gels were increased with increased ALGMA amount. According to the authors, this change happened because of the hydrogen bonds which were created by the ALGMA, and these bonds supported IPN structure in the hydrogels. Moreover, they support their rheology findings with FTIR results. FTIR study of the alginate showed a peak at 3440 cm^−1^ in the hydrogen bond region, and then this peak shifted to 3275 cm^−1^ for PEGMA/NIPAm/ALGMA hydrogels. Based on these findings, Zhao et al. interpreted the shift as formation of hydrogen bonds between the PEGMA/NIPAm/ALGMA, and this was attributed to IPN formation in the gel [105].

### 3.2. Drug Delivery Hydrogels

Darge et al. designed a sodium alginate and sulfobetaine methacrylate (SBMA) monomer-based IPN hydrogel to produce microneedles (MN) for drug delivery into the dermal interstitium across the stratum corneum. Lipopolysaccharide (LPS) and doxorubicin (DOX) were loaded into MN arrays for use as an immunochemotherapy agent.

IPN hydrogel was synthesized by using the sequential IPN synthesis method. First, SBMA was chemically crosslinked by photo crosslinking, and it formed a polymer network. Then, alginate was ionically crosslinked by using calcium ions, and it created the second network. These two networks together formed a SBMA/Alginate IPN hydrogel.

FTIR analysis and mechanical testing were utilized for characterization of the IPN hydrogel. In FTIR spectra, the signal at 1720 cm^−1^ in the SBMA monomer was seen in IPN hydrogel as well. This point indicates that monomers created a network by chemically crosslinking. On the other hand, the COO^−^ signal of sodium alginate was seen at 1595 cm^−1^, then the signal shifted to 1642 cm^−1^ in the IPN hydrogel. This change indicates the ionic crosslinking for alginate via calcium ions. According to these findings, it was interpreted that there is an IPN hydrogel formation [106].

A universal testing machine was used to obtain mechanical testing results of hydrogels. They examined different synthesis methods on hydrogels. In this article, three synthesis methods were used. These are (i) photo crosslinking, to create SBMA networks, (ii) ionic crosslinking, to get alginate networks, and (iii) photoionic crosslinking, to form IPN hydrogels. By using photo crosslinking, the highest tensile strength was achieved. However, its strain was very low compared to others. Therefore, it was confirmed that the hydrogels that formed by only photo crosslinking method had a brittle and rigid structure. On the other hand, by only using the ionic crosslinking method, the hydrogels had the maximum strain and minimum tensile strength. A soft structure is obtained by this method. Finally, by using the photoionic crosslinking method, desired strength and strain results were achieved. Based on these results, it can be interpreted that the sequentially formed IPN got the properties from both alginate and SBMA networks [106].

**Figure 9:**
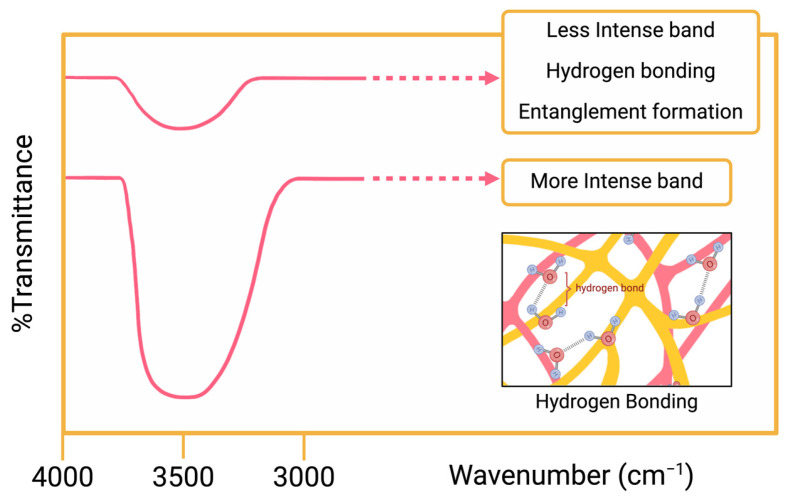
Illustration of different intensity bands at hydrogen bond region (made by Biorender.com).

Agnihotri et al. synthesized a novel hydrogel to use in controlled drug delivery. The aims of this study were (i) sustaining a stable blood level, (ii) reducing negative effects of drugs, and (iii) increasing the drug efficacy. To achieve their goals, they formed an IPN microsphere hydrogel from gellan gum (GG) and poly(vinyl alcohol) (PVA) via emulsion crosslinking method. Carvedilol, which is a hypertension treatment drug, was loaded to the microspheres to understand drug release kinetics and glutaraldehyde (GA) crosslinker was used for different crosslinking levels. FTIR, DSC and tensile strength measurements were used to observe interpenetrating network of the microspheres. In the FTIR interpretations, it was observed that GG’s broad band at 3444 cm^−1^ and PVA’s broad band at 3425 cm^−1^. Unloaded GG-PVA microspheres showed their broad band at 3487 cm^−1^. Their values were similar; however, the intensities of the bands showed differences. Figure 9 shows a similar illustration of the intensities of the bands at the hydrogen bond region. Unloaded GG-PVA microspheres had less intensity in contrast to GG and PVA. It was determined that these data were observed because uncrosslinked hydroxyl groups of the glucopyranose ring bonded with hydrogen and created hydrogen bonding [107]. As mentioned at the beginning of this article, physical crosslinking occurs to form IPNs in materials. According to Agnihotri et al., this finding confirms the IPN structure of the microspheres.

Another approach to confirm the hydrogel’s IPN structure is via DSC thermograms. The endothermic peaks of the GG, PVA, and unloaded GG-PVA microspheres were compared. The endothermic peak of the GG was 248 °C. PVA had two endothermic peaks, and they were 210 °C and 290 °C, respectively. The unloaded GG-PVA microspheres showed two peaks. The first one was at 224 °C, which is between the values of GG and PVA, and the second one was at 320 °C, which is a higher value compared to GG and PVA. The interpretation of these findings leads to the conclusion that unloaded GG-PVA microspheres formed a more crystalline polymer structure. As a result, the higher endothermic peaks (which are a sign of high melting temperature) of unloaded GG-PVA indicated that there was an IPN formation because IPN needs higher temperatures to melt compared to single-network structures.

Last but not least, tensile strength measurements were conducted to understand if there was an IPN structure in the microspheres. The increased amount of GA in the synthesis of the microspheres resulted in an increase in strength. When the GG and microspheres were compared, the microspheres showed five to six times higher tensile strength. The researchers interpreted this finding as a confirmation of the IPN structure [107].

Mohamadnia et al. focused their studies on pH-sensitive interpenetrating hydrogel beads to control drug delivery. Some responsive properties, such as pH, temperature, and ionic strength, were used to control the swelling behavior of the beads. Beads were synthesized from two different biopolymers: alginate and carrageenan. Betamethasone acetate, a kind of corticosteroid drug, was loaded into the beads to examine their release behaviors. Also, to enhance the swelling properties, an IPN structure was formed in the hydrogel beads. SEM was used to observe the IPN structure of the hydrogel beads in this study. According to Mohamadnia et al., the interpenetrating network of the beads was the cause of such a unique structure. Loaded beads looked more swollen and orderly due to the encapsulation of the drug into the IPN entanglements [108].

Bhattacharya et al. synthesized sodium carboxymethyl cellulose and sodium carboxymethyl xanthan gum-based (SCMC-SCMXG) IPN hydrogel beads via an emulsion gelation process. They loaded the beads with diclofenac sodium (DS), which is an anti-inflammatory drug, to observe the drug release mechanism. XRD analysis of the beads was carried out to investigate their IPN structure. According to Bhattacharya et al., the drug’s crystalline form shifted to an amorphous form in the beads [109] because the drug had been distributed into the entanglements of the IPN structure. They associated the crystallinity change with the IPN structure and found proof to confirm IPN structure in the hydrogel beads.

Pan et al. formed an IPN hydrogel that contained sugar bagasse cellulose (SBC), carboxymethylcellulose (CMC), and poly(N-isopropylacrylamide) (PNIPAm). Epichlorohydrin was added as a crosslinker. The hydrogel was intended to be used as a smart drug delivery system. SEM figures of the SBC-CMC hydrogel and the IPN hydrogel can be found in the paper by Pan et al. In their SEM figures, the SBC-CMC hydrogel displayed a porous form. However, the IPN hydrogel showed a net-like structure around the first porous form. According to Pan, this confirms the IPN structure of the hydrogel. Additionally, FTIR analyses of the hydrogels provided results to prove that its structure formed an IPN. In the FTIR studies, there was no significant peak shifting between the crosslinked SBC-CMC hydrogel and IPN hydrogel. Their peaks were reasonably similar to each other, so it was inferred as there was no chemical bonding between them and they were just entangled [110].

Kim et al. (2018) worked on a temperature- and pH-sensitive IPN hydrogel which consisted of hyaluronic acid (HA) and poly (N-isopropylacrylamide) (PNIPAM). The injectable hydrogel was created for transdermal delivery of luteolin as psoriasis skin relief. PNIPAM and HA were used for temperature sensitivity and pH sensitivity, respectively. Kim et al. created the first network by radical polymerization of PNIPAM and with the HA the gel structure formed a semi-IPN. The second network was formed by Michael addition for HA. As a result, a full IPN structure was achieved in the study. According to swelling ratio studies, the full IPN hydrogel had a denser structure than the semi-IPN hydrogel. Also, studies on the swelling rate change by pH showed that the acidic setting supported intramolecular hydrogen bond formation and reduced swelling of hydrogen, which can be attributed to IPN formation in the hydrogel [111].

### 3.3. Hydrogels for Other Biomedical Applications

Wahid et al. aimed to prepare a hydrogel with advanced mechanical and antibacterial features to be used in the biomedical field. Bacterial cellulose (BC), chitosan (CS), and glutaraldehyde crosslinker were used to synthesize semi-IPN hydrogels. In this manner, the BC’s properties, such as a great amount of water retention, biocompatibility, biodegradability, high porosity, and nontoxicity, were combined with chitosan’s antibacterial properties to obtain a biomedically applicable material. Characterization of the hydrogels was carried out with FTIR. In the FTIR analysis, new peaks occurred at 1705 cm^−1^ only for CS-containing hydrogels. This peak was explained by glutaraldehyde’s C=O of the aldehyde group. Moreover, imide bond formation was observed for CS at 1558 cm^−1^ in the presence of glutaraldehyde. These peaks indicate that the CS interacted with glutaraldehyde. On the other hand, OH and NH_2_ shifting were detected for CS, while H-O-H shifting was detected for BC [34]. These lower wavenumbers are interpretable as caused by hydrogen bond formation between BC and CS. As a result, it was understood that CS chains linked with other CS polymer chains with glutaraldehyde while BC polymer chains entangle to them via hydrogen bonding. In conclusion, it was confirmed that the BC-CS hydrogels had a semi-IPN structure [34].

The presence of hydrogen bonding was also examined with XRD analysis. In these studies, CS hydrogel showed an amorphous structure at ~20°. Normally, BC has a crystalline structure because of the hydrogen bonding between its layers. However, it showed a wide and less intense peak at ~20°, in the form of BC-CS hydrogel. This was observed because BC’s hydrogen bonds were entangled with the CS polymer chains instead of its own layers, which caused it to alter its structure from crystalline to amorphous. Therefore, this situation indicates that a semi-IPN structure was formed in the hydrogel.

Another characterization method to reveal the IPN structure was FESEM analysis. Only CS-containing hydrogel and BC-CS-containing hydrogels (with different ratios of BC) were examined under FESEM. CS hydrogel showed big pores. However, BC-CS hydrogels’ pore size was reduced when the amount of BC increased in the hydrogel (BC-20 and BC-40). Increased network structure occurred due to entanglements, so this confirms that the entanglement of the BC to the CS and semi-IPN structure [34].

Sampath et al. prepared a cellulose nanocrystal (CNC) reinforced chitosan (CS) hydrogel with the support of glutaraldehyde crosslinker. This hydrogel was defined as a semi-IPN hydrogel due to FTIR analysis [112].

FTIR analyses were conducted for CNC, CS, CS, and CNC-CS hydrogels to confirm the semi-IPN structure of the hydrogel. Both CNC and CS hydrogel peaks were observed in the CNC-CS hydrogels. There was not an extreme amount of shifting in the peak of the CNC-CS hydrogels. According to Sampath, this means that CNCs were physically bonded to the CS hydrogel, leading to the determination of the semi-IPN structure [112].

Tığlı and Gümüşderelioğlu developed a cartilage scaffold from alginate (Alg) and chitosan (CS). They synthesized this Alg-CS semi-IPN scaffold by freeze-drying. Calcium chloride (CaCl_2_) crosslinker was used to synthesize the alginate scaffold. SEM and fluorescence microscope images were analyzed to see the scaffold’s semi-IPN structure. The Alg-CS (70:30) % (*v*/*v*) scaffold shows that the chitosan settled into the alginate scaffold. On the other hand, to see this network formation clearly, fluorescence microscope images were used.

According to figures in Tığlı and Gümüşderelioğlu’s studies, chitosan is marked with fluorescein isothiocyanate. The bright sections were chitosan, which collapsed in the alginate scaffold. Distribution of the chitosan was shown in its exact location with the help of the fluorescence microscope [113].

### 3.4. Hydrogels for Non-Biomedical Applications

Due to the toxic behavior of azo dyes in the environment, Ngwabebhoh et al. developed a superabsorbent material to enhance the wastewater treatment process. They synthesized chitosan/starch semi-IPN hydrogels to achieve improved adsorptive removal of the dye Direct Red (DR80) from an aqueous medium. Glutaraldehyde was used as a crosslinking agent. FTIR analyses were conducted to characterize the structure of their material. The band observed at 1644 cm^−1^ for hydrogel indicates that the crosslinking process via glutaraldehyde was achieved with only chitosan. Also, hemiacetal and acetal bond formations between starch and glutaraldehyde made unsteady interactions with each other. Thus, it was concluded that starch has physical interactions with crosslinked chitosan in the hydrogel. As an inference, the hydrogels were classified as semi-IPN [9] because it is known that if one polymer crosslinks and the second one entangles the first polymer, it is called a “semi-IPN”. According to study, chitosan chains were crosslinked with each other. However, starch polymers were seen to be entangled with them.

## 4. Discussion

In this review article, FTIR, Raman, SEM, FESEM, DSC, XRD, DMA and rheology methods were examined for confirming IPNs in hydrogels.

While gathering the literature from different articles, it was observed that the great majority of the authors did not give concrete results about how they determined the IPN structure in their material. However, it is important to provide confirming data to say if the material is IPN or not because IPNs’ definition can be easily confused with polymer blends. It is useful to conduct different characterization methods to obtain information about a hydrogel’s internal structure.

The articles that were chosen from the literature mentioned how they confirmed IPN structure with characterization methods in their studies (Table 1). There is not only one characterization method to confirm networks in a hydrogel, and there is not only one type of evidence to confirm the structure. All the characterization processes of IPN depend upon the experiment method, the material’s nature, and the researchers’ experience with the literature and truly interpreting these results.

It is seen that the most used detection method was FTIR. This method gives the researcher the most relatable results regarding hydrogel IPN structure. The chemical bonds and physical interactions between the networks are identified with this technique. Also, it is not an invasive method for materials and does not alter the sample’s structure.

XRD and DSC give information about a material’s amorphous and crystalline structure. DSC can be used in samples that have different structures from each other. If a material consists of these two different arrangement types (amorphous and crystalline), DSC can be used. However, DSC may not be suitable for more than two networks due to the complexity of the identification process. Also, it is a destructive method.

Mechanical testing methods indicate a material’s mechanical properties. Stress–strain curves of the base materials and the IPN hydrogel are compared with each other to determine IPN structure. Rheology method is a mechanical testing method. The storage moduli of the base and IPN samples are compared to each other to find a relatable connection between them. Rheology does not give a chemical viewpoint to the material, so the entanglement theory cannot be completely confirmed by using only this method.

SEM and FESEM are used to obtain an image of the material’s morphology. These methods require a vacuum environment, so hydrogels must be prepared for this condition. The preparation process can cause structural changes within the hydrogel. These methods give the clearest structural identification for the samples, but the most important point here is the accurate interpretation and experience of the researcher within the field. In some cases, entanglements may not be seen with these techniques.

## 5. Conclusions

As a result, FTIR was found to be the most promising method for determining IPN structures in hydrogels. Other characterization methods are also useful for confirming the structure; however, they need much more effort, experience, and clearness for their interpretations. These methods can be used together to confirm the IPN structure, and a combination of these methods would give even more accurate results. It is feasible to use more than one method and gain experience to correctly interpret the results. In the future, as studies on IPN hydrogel increase, they will provide more information and data about the accuracy of these determining methods.

## Figures and Tables

**Table 1:** Studies analyzed in this article.

Author/Year	Materials	Material Form	IPN Type	Application	Properties	Characterization Methods to Determine IPN Structure
Darge et al., 2022 [106]	Sodium alginate and sulfobetaine methacrylate (SBMA)	Microneedles	Sequential IPN	Drug delivery	Desired immunochemotherapy agent release Suitable mechanical properties for drug delivery	FTIR Mechanical Testing
Agnihotri & Aminabhavi, 2005 [107]	Gellan gum (GG) and poly(vinyl alcohol) (PVA)	Microspheres	IPN	Controlled drug delivery of a hypertension drug	Enhanced entrapment of drug into networks Smooth microsphere surface	FTIR DSC Mechanical Testing
Mohamadnia et al., 2007 [108]	Alginate and carrageenan	Beads	IPN	Drug delivery of a corticosteroid drug	Controllable drug release with pH, temperature and ions	SEM
Bhattacharya et al., 2013 [109]	Sodium carboxymethyl cellulose and Sodium carboxymethyl xanthan gum (SCMC-SCMXG)	Beads	IPN	Drug delivery of an anti-inflammatory drug	Successful drug release in alkaline medium Reduced aftereffect for gastritis	XRD
Wahid et al., 2019 [34]	Bacterial cellulose (BC), chitosan (CS)	Slurry	Semi IPN	Antimicrobial applications	High mechanical and thermal stability Antibacterial	FTIR XRD FESEM
Sampath et al., 2017 [112]	Cellulose nanocrystal (CNC) and chitosan (CS)	Gel	Semi IPN	Biomedical applications	Enhanced mechanical properties pH sensitivity	FTIR
Pan et al., 2018 [110]	Sugar bagasse cellulose (SBC), carboxymethylcellulose (CMC) and poly(N-isopropylacrylamide) (PNIPAm)	Gel	IPN	Drug delivery	Sensitivity to pH and temperature	SEM
Tığlı & Gümüşderelioğlu, 2009 [113]	Alginate (Alg) and chitosan (CS)	-	Semi IPN	Cartilage scaffold	Support proliferation Maintain cell functionality	SEM Fluorescence microscope
Wang et al., 2017 [100]	p(*N*, *N*-dimethyl- acrylamide-*co*-4-acryloyldopamine), p(*N*, *N*-dimethylacrylamide-*co*-3-acrylamidopheynlboronic acid) and p(*N*, *N*-dimethylacrylamide-*co*-4-acryloyloxybenzaldehyde)	Gel	Semi IPN	3D printing	Tunable mechanical properties by pH and solid content Self-healing in both acidic and basic environment Injectable	Rheology
Shariatzadeh et al., 2021 [101]	Gelatin methacrylate (GelMA) and hyaluronic acid (HA)	Cryogel	Sequential IPN	Soft tissue engineering	Highly porous Hydrophile Shape memory Injectable	FTIR
Cui et al., 2023 [102]	Oxidized alginate (OSA)/gelatin (Gel)/ cellulose nanofiber (CNF)	Gel	Semi IPN	Bone repair	Self-healing Tunable water uptake capacity Tunable degradation and swelling behavior Injectable	FTIR
Mu et al., 2020 [103]	Injectable platelet-rich fibrin (iPRF) and gelatin nanoparticles (GNPs)	Gel	IPN	Bone healing	Mechanically tough Bioactive Self-healing Degradable Injectable	SEM
Kim et al., 2018 [111]	Hyaluronic acid (HA)/poly (N-isopropylacrylamide) (PNIPAM)	Gel	Full IPN	Transdermal drug delivery	pH and temperature sensitive	Swelling ratio
Zhao et al., 2014 [105]	Poly(ethylene glycol) methacrylate (PEGMA)/ N-isopropylacrylamide (NIPAm)/ methacrylated alginate (ALGMA)	Gel	IPN	Release studies	Injectable Temperature and pH sensitive	Rheology FTIR
Ngwabebhoh et al., 2016 [9]	Chitosan and Starch	Gel	Semi IPN	Wastewater treatment	Efficiently elimination of dye from aqueous solution Non-toxic Eco-friendly	FTIR

## Data Availability

No new data were created.
